# Impact of intraoperative goal-directed therapy on perioperative outcomes in kidney transplantation: a multicenter randomized controlled trial

**DOI:** 10.1007/s11739-025-04021-2

**Published:** 2025-06-25

**Authors:** Laici Cristiana, Gamberini Lorenzo, Vitale Giovanni, Guizzardi Chiara, Ravaioli Matteo, La Manna Gaetano, Comai Giorgia, Skurzak Stefano, Cerutti Elisabetta, Di Blasi Salvatore, Cerchiara Paolo, Gobbi Fabio, Cimatti Mirella, Ramahi Linda, Siniscalchi Antonio

**Affiliations:** 1 Postoperative and Abdominal Organ Transplant Intensive Care Unit, IRCCS Azienda Ospedaliero-Universitaria di Bologna, Bologna, Italy; 2 Department of Anaesthesia, Intensive Care and Prehospital Emergency, Ospedale Maggiore Carlo Alberto Pizzardi, Bologna, Italy; 3 Internal Medicine Unit for the Treatment of Severe Organ Failure, IRCCS Azienda Ospedaliero-Universitaria di Bologna, Bologna, Italy; 4 Department of Medical and Surgical Sciences (DIMEC), Alma Mater Studiorum-University of Bologna, Bologna, Italy; 5 Hepatobiliary and Transplant Surgery Unit, IRCCS Azienda Ospedaliero-Universitaria di Bologna, Bologna, Italy; 6 Nephrology, Dialysis and Kidney Transplant Unit, IRCCS Azienda Ospedaliero-Universitaria di Bologna, Bologna, Italy; 7 SC Anestesia e Rianimazione 2 AOU Città della Salute e Della Scienza, Turin, Italy; 8 Department of Anesthesia, Transplant and Surgical Intensive Care, Azienda Ospedaliero Universitaria delle Marche, Ancona, Italy; 9 Department of Anesthesia and Intensive Care, San Luigi Gonzaga Hospital, Orbassano, Italy; 10 Department of Anesthesia and Intensive Care, Humanitas Gradenigo Hospital, Turin, Italy

**Keywords:** Kidney transplantation, Goal-directed therapy, Length of stay, Postoperative complications, Non-invasive monitoring

## Abstract

Appropriate fluid management is crucial in anesthesiologic management during kidney transplantation (KT). Traditional parameters such as blood pressure and central venous pressure are unreliable and weakly supported by guidelines. Goal-directed fluid therapy (GDT) has emerged as a technique for administering fluids and vasoactive drugs based on algorithms to ensure adequate tissue perfusion. Current data suggest GDT may reduce tissue edema and respiratory complications in KT recipients. This multicenter, single-blind randomized controlled trial compared conventional fluid management strategies with a GDT algorithm using non-invasive pulse pressure contour analysis monitoring (ClearSight®) in KT patients. The primary outcome was the hospital length of stay. Secondary outcomes included postoperative complications, delayed graft function, 90-day graft loss, and intensive care unit (ICU) length of stay. Patients and postoperative care physicians were blinded to group assignments. The study enrolled 181 KT recipients over 32 months. The hospital length of stay did not significantly differ between the groups, with a difference of 0.5 days (95% CI: -2.5 to 5 days). No significant differences were found in surgical and medical complications, delayed graft function, graft loss, or ICU length of stay. In KT recipients, using a GDT algorithm did not result in clinically meaningful differences in hospital stay, complications, or graft dysfunction/loss.

## Introduction

The rates of kidney transplantation (KT) across Europe have significantly increased over the past decade, mainly due to the adoption of expanded criteria donors, early referral of potential donors, and crossover donation programs [1]. Despite these advancements, postoperative medical and surgical complications remain common and can adversely affect early graft survival [2].

Effective perioperative fluid management is crucial for reducing complications in surgical patients, especially during high-risk procedures like KT [3].

Central venous pressure (CVP) values have traditionally guided fluid management during KT. However, current guidelines indicate that using CVP as a guide for fluid administration is only weakly supported [4, 5]. Excessive fluid administration can damage the endothelial glycocalyx and lead to fluid overload, increasing the risk of fluid shifting into the third space and causing organ failure [6].

Over the past three decades, advanced hemodynamic monitoring has expanded to include additional variables such as cardiac index (CI) and oxygen delivery index (DO_2_I), which are critical for ensuring adequate organ perfusion and reducing postoperative complications and organ dysfunctions [7].

Goal-directed therapy (GDT) is a fluid management strategy that employs inotropes and/or vasopressors based on predefined algorithms to achieve target values of advanced hemodynamic variables [8]. GDT has been suggested as an effective method to reduce postoperative complications in surgical patients [9].

Currently explored advanced hemodynamic monitoring techniques for KT include arterial waveform analysis [8, 10–12] and transesophageal Doppler (TED) monitoring [13, 14], while other less-studied techniques still used for other major surgeries are transesophageal echocardiography and bioimpedance-based technologies [15].

A recent systematic review and meta-analysis of interventional studies demonstrated that GDT significantly reduces the incidence of tissue edema and respiratory complications in KT recipients [16].

Moreover, although statistical significance was not achieved, patients receiving cadaveric grafts exhibited lower serum creatinine levels on postoperative days 1 and 3. Additionally, patients monitored with arterial waveform analysis devices experienced a lower incidence of postoperative hemodialysis [16].

Non-invasive cardiac output (CO) monitoring has gained attention in the perioperative management of KT patients due to its potential to improve fluid management without the risks associated with invasive techniques [17].

These monitors, such as pulse contour analysis and bioimpedance-based systems, provide continuous real-time data on hemodynamics This is crucial given the delicate balance required to optimize organ perfusion while avoiding fluid overload in these patients.

The ClearSight® system (Edwards Lifesciences, Irvine, California, USA) is an advanced non-invasive pulse contour-based system, allowing for continuous arterial blood pressure measurement using finger cuff technology. It performs pulse pressure contour analysis by applying advanced vascular unloading technology. This method allows analyzing the contour of the arterial pressure waveform to reliably derive advanced hemodynamic parameters such as CO, stroke volume (SV), and systemic vascular resistance (SVR), providing valuable data for optimizing fluid management and overall patient care [18].

To date, little is known about the potential role of GDT based on non-invasive hemodynamic monitoring on major patient and graft-related outcomes. Therefore, this study was conducted to answer the following questions: (1) Does non-invasive hemodynamic monitoring-based GDT decrease the overall hospital stay in KT patients? (2) Does this technique reduce ICU stay, postoperative complications, and increase graft and patient survival?

## Methods

We conducted a multicenter, single blind randomized controlled trial (RCT) comparing two groups of patients undergoing single or dual KT from deceased donors. All eligible patients were randomized via a computer-generated randomization list to either the intervention group (GDT) or the control group (conventional fluid strategy).

In the GDT group, patients underwent minimally invasive continuous CI monitoring, and the protocol flowchart guided interventions to achieve specific hemodynamic goals. In the control group, corrective actions were guided by conventional hemodynamic parameters according to good clinical practice and international guidelines.

The primary outcome was the overall length of stay in the hospital. The secondary end points were the incidence of postoperative surgical and medical complications, ICU length of stay, and 90-day graft survival. Additionally, 1-year patient survival was investigated as a non-preplanned secondary outcome.

Patient data were collected prospectively by designated researchers using a digital case report form.

The study protocol received approval from the Institutional Review Board of the participating hospitals: IRCCS Azienda Ospedaliero-Universitaria di Bologna, Azienda Ospedaliero-Universitaria delle Marche of Ancona, and Città della Salute e della Scienza of Torino. It was also registered on ClinicalTrials.gov (NCT05035537) on August 29, 2021. The registration was delayed due to organizational factors and, subsequently, the coronavirus disease 2019 (COVID-19) pandemic.

The inclusion criteria were age ≥ 18 years, single or dual KT from a cadaveric donor, American Society of Anesthesiologists (ASA) class III-IV, and positive expression of consent.

Exclusion criteria were any ongoing arrhythmia or history of arrhythmias, previous organ transplantation, transplantation from a living donor, combined KT with any other organ, and any situation where the potential need for advanced hemodynamic monitoring was anticipated.

After inclusion, patients were randomized 1:1 to either the control group (conventional management) or the GDT group.

### Perioperative management

In the intraoperative phase, standard monitoring was the same for both groups: electrocardiogram, peripheral oxygen saturation (SpO2), non-invasive blood pressure (NIBP) or invasive blood pressure (IBP), capnography, and bispectral index (BIS). All enrolled patients underwent general anesthesia with orotracheal intubation. Induction was achieved with propofol (2 mg/kg) and fentanyl (1.5 mcg/kg), and after reaching a BIS value < 60, a bolus of rocuronium (0.6 mg/kg) or cisatracurium (0.2 mg/kg) was administered. Sevoflurane 0.8–1 minimum alveolar concentration (MAC) and fentanyl 0.8–1 mcg/kg/h were used for maintenance. Mechanical ventilation was set with a tidal volume of 6–8 mL/kg and respiratory rate adapted to a target ETCO2 of 35–45 mmHg, FiO2 was set to keep SpO2 > 96%, and positive end-expiratory pressure (PEEP) to 5 cmH2O. A transversus abdominis plane (TAP) block was ultimately administered for postoperative pain relief, according to individual judgment.

In all patients, a central line was placed after induction. Patients randomized to the GDT group underwent ClearSight® monitoring, while those randomized to the conventional strategy group received traditional invasive blood pressure monitoring (IBP) through a 20-G arterial radial catheter.

All patients received a standard dose of immunosuppressant at induction as per the single center’s practice and a corticosteroid bolus (methylprednisolone 250 mg) before each graft reperfusion (methylprednisolone 250 mg, two boluses if a dual KT was performed). All patients were admitted to the ICU at the end of the surgery.

### Hemodynamic management

Baseline fluid therapy consisted of 0.5 mL/kg/h crystalloids for both groups. Both groups'mean arterial pressure (MAP) was ≥ 70 mmHg. In the control group, conventional static hemodynamic parameters (CVP and IBP) were used to guide corrective actions (fluids, vasoactive agents) according to good clinical practice and international guidelines, to maintain this target [4].

**Fig. 1:**
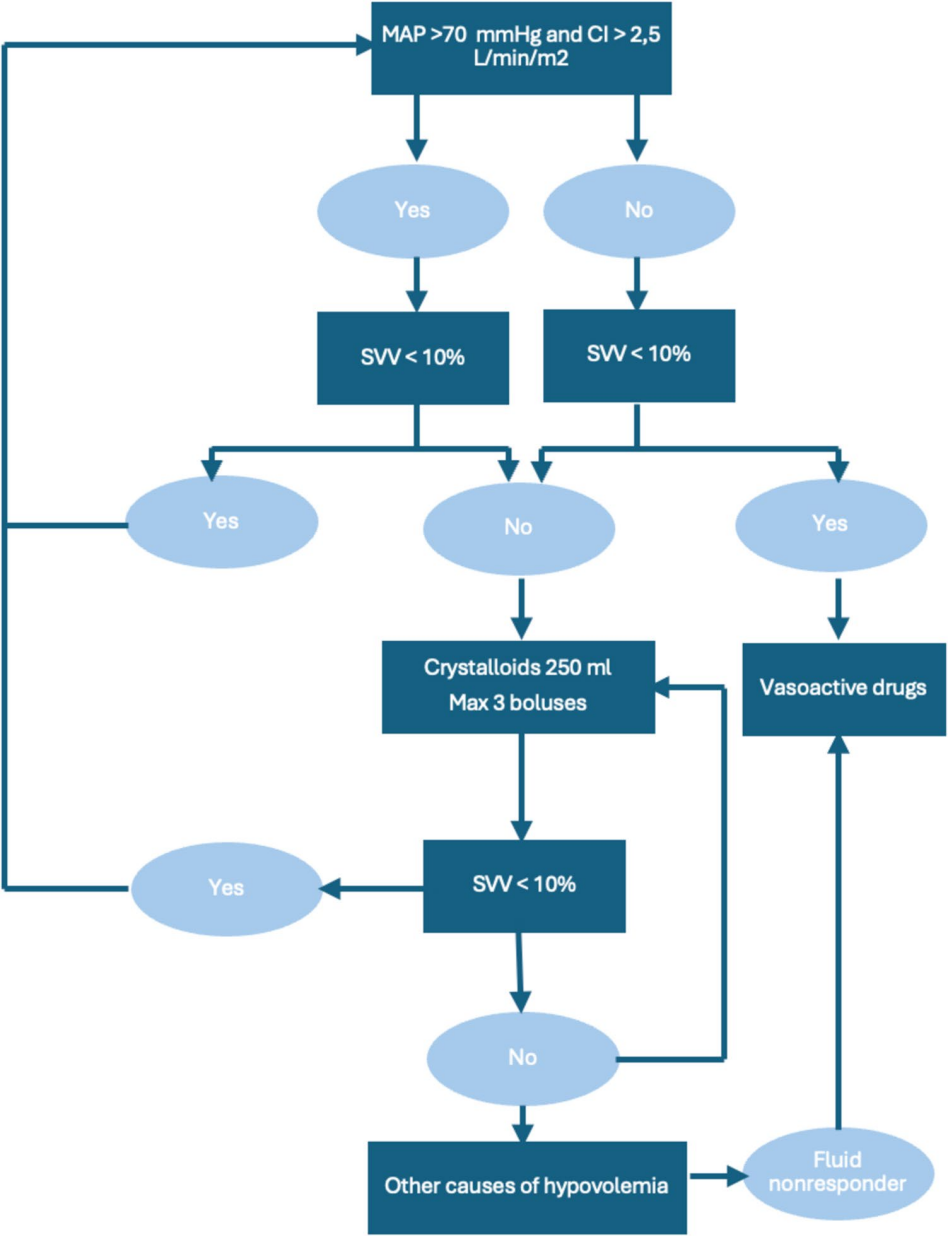
Goal-directed therapy diagram. *MAP* mean arterial pressure, *CI* cardiac index, *SVV* stroke volume variation

In the GDT group, CI ≥ 2.5 L/min/m2 and SVV < 10%, measured by ClearSight®, were added to the MAP target. The administration of additional fluid boluses and the introduction of vasoactive drugs were guided by a standard algorithm shown in Fig. 1. When the MAP, CI, and SVV values were equal to or above the target, baseline infusion was maintained. In case of SVV > 10%, a 250 mL fluid bolus was administered, up to a maximum of three boluses. Finally, in case of CI < 2.5 L/min/m2 despite SVV being < 10%, vasoactive drugs were introduced. After achieving the target values for the hemodynamic parameters, reevaluations were conducted every 15 min.

Fluid boluses were crystalloids; albumin was only allowed if preoperative serum albumin was < 2.5 g/dL; patients were transfused packed red blood cells (RBC) in case of hematocrit (Hct) < 24% or Hb < 80 g/L. In the ICU, all patients were managed using the same protocol, receiving a standard infusion of crystalloids (0.5 mL/kg/h) and a potential continuous infusion of furosemide ranging from 5 to 20 mg/kg/h targeted to maintain a urinary output of 0.3–0.5 ml/kg/h. The infusion was stopped when it reached 1 ml/kg/h.

### Discharge criteria

Patients were discharged from the ICU after complete weaning from mechanical ventilation and vasoactive drugs, and after the absence of signs suggesting cardiovascular complications or infections. Hospital discharge was allowed if the following targets were met: stability of hemodynamic parameters, lack of medical or surgical complications, and withdrawal from dialysis. The physicians involved in the patient discharge decision were blinded to group allocation.

### Collected data

Perioperative collected data were age, sex, body mass index (BMI), ASA class, baseline comorbidities and organ dysfunctions, volumes and types of fluids infused intraoperatively and during the first 24 h post-surgery, and the need for any vasoactive drug.

Adherence to GDT was assessed by the percentage of intraoperative time spent within the target thresholds for MAP, CI, and SVV.

The follow-up collected data were postoperative pulmonary, cardiovascular, and infectious complications reaching a Clavien–Dindo grade ≥ II during the first five postoperative days.

Moreover, any Clavien–Dindo grade ≥ III complication happening within postoperative day 30 and hospital and ICU length of stay were recorded. Graft-specific collected follow-up data were the development of delayed graft function (DGF), defined as the need for renal replacement therapy within the first seven postoperative days [19], and 90-day graft loss, defined as the failure of the transplanted kidney, needing a return to dialysis or re-transplantation.

### Sample size calculation and preplanned statistical analysis

The sample size was calculated according to the primary outcome and considering an estimated average hospital length of stay in the standard therapy group of 15 ± 7 days, and a decreased mean of 12 days (20%) in the experimental group. The estimated sample size for a two-tailed independent samples t-test, setting an alpha error of 0.05, power 85%, and 1:1 enrollment ratio, was 200 patients.

The personnel conducting data cleaning and statistical analyses were blinded to patients'group assignment. Continuous variables, according to the normality of their distribution, explored with QQ plots, were expressed as means and standard deviations (SD) or median and interquartile range (IQR) and then compared using an independent samples *t*-test or the Mann–Whitney *U* test accordingly. Categorical variables were expressed as numbers and percentages, and the eventual differences between groups were evaluated through the Chi-square test and Fisher’s exact test. The median difference in hospital length of stay between the groups was calculated using bootstrap resampling. Specifically, 1000 bootstrap samples were generated to estimate the distribution of the median difference.

Graft survival was calculated from the day of surgery to 90 days and 1 year. Moreover, survival curves were created and compared with the log-rank test. Statistical significance was defined by a p-value ≤ 0.05, and all the tests were two tailed. All the analyses were performed through R Core Team (2024) version 4.4.0.

## Results

Recruitment for the study began in January 2018 and concluded in November 2022. The study was temporarily suspended from March 2020 to May 2022 due to the COVID-19 pandemic. Follow-up activities were completed in November 2023.

Out of 200 patients assessed for eligibility, 15 did not meet the inclusion criteria and 4 declined to participate. Due to time constraints specified in the protocol, the study ended before reaching the target sample size, enrolling 181 out of the desired 200 patients (91%).

**Fig. 2:**
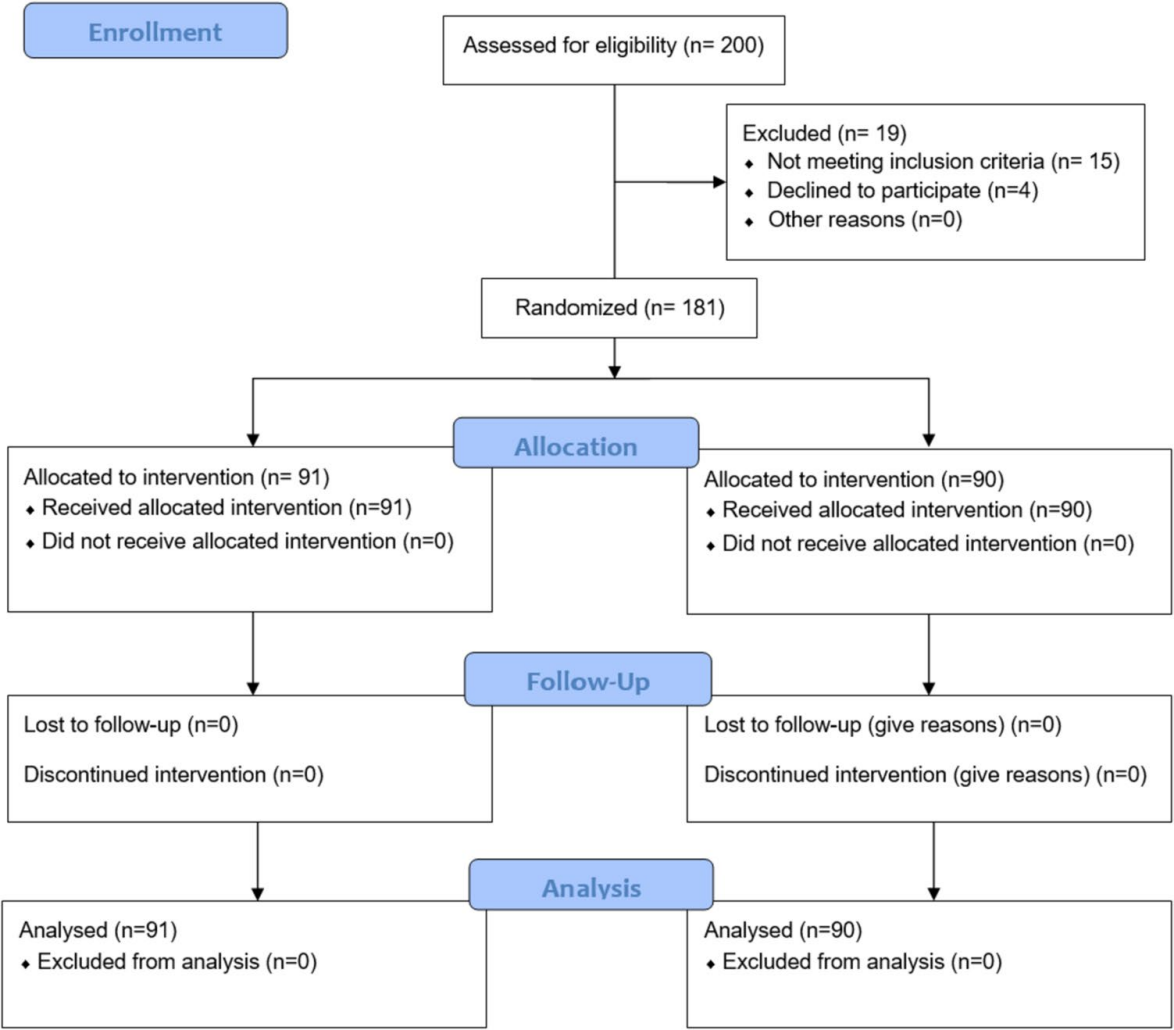
CONSORT diagram

Of the 181 patients enrolled, 91 were allocated to the GDT group and 90 to the conventional fluid therapy group. The CONSORT diagram (Fig. 2) visually represents patient flow through the study.

Baseline characteristics were comparable between the groups (Table 1). The average age was 51 years in the GDT group and 52 years in the conventional fluid therapy group (*p* = 0.642). The proportion of female patients was 39.6% (36 out of 91 cases) in the GDT group and 37.8% (34 out of 90 cases) in the conventional group (*p* = 0.925). BMI was similar in both groups, with a median of 24.2 kg/m^2^ (IQR 21.6–26.3) in the GDT group and 24.29 kg/m^2^ (IQR 22.8–26.4) in the conventional fluid group (*p* = 0.646). There were no significant differences in the distribution of ASA class, comorbidities, smoking status, and renal replacement therapy between the two groups.

**Table 1:** General characteristics of the patients enrolled

	Goal-directed therapy *n* = 91	Conventional therapy *n* = 90	*p*
Age—years, mean (SD)	51.00 (13.09)	51.86 (11.91)	0.642
Sex—female, n (%)	36 (39.6)	34 (37.8)	0.925
Body mass index—kg/m^2^, IQR	24.22 [21.6—26.3]	24.29 [22.8—26.4]	0.646
Preoperative Hb—g/dL, mean (SD)	11.93 (1.57)	11.88 (1.65)	0.839
ASA class, n (%)			0.987
3	87 (95.6%)	86 (95.6%)	
4	4 (4.4%)	4 (4.4%)	
Comorbidities, n (%)			
Pulmonary disease	23 (25.3%)	19 (21.1%)	0.626
Cardiovascular diseases	76 (83.5%)	78 (86.7%)	0.699
Central nervous system diseases	11 (12.1%)	9 (10.0%)	0.833
Diabetes	8 (8.8%)	6 (6.7%)	0.797
Other	38 (41.8%)	44 (48.9%)	0.415
Smoke status, n (%)			0.480
Never smoked	63 (69.2%)	58 (64.4%)	
Current smoker	15 (16.5%)	13 (14.4%)	
Former smoker	13 (14.3%)	19 (21.1%)	
Renal replacement therapy, n (%)			0.890
None	2 (2.2%)	3 (3.3%)	
Hemodialysis	73 (80.2%)	72 (80.0%)	
Peritoneal dialysis	16 (17.6%)	15 (16.7%)	

*SD* standard deviation, *IQR* interquartile range, *ASA* American Society of Anesthesiologists

Donor characteristics and intraoperative variables, described in Table 2, were also similar between the groups. Donation after brain death occurred in 82.4% of cases in the GDT group and 80.0% in the conventional fluid group (*p* = 0.821). Intraoperative vasopressors were used in 19 (19.8%) patients in the GDT group compared to 16 (17.8%) in the conventional fluid group (p = 0.877). The total volume of intraoperative fluids administered was similar between the groups, with a median of 6.65 mL/kg/h (IQR 4.23—9.63) in the GDT group and 6.41 mL/kg/h (IQR 4.67—9.05) in the conventional group (*p* = 0.869).

**Table 2:** Intraoperative data and hospital stay

	Goal-directed therapy *n* = 91	Conventional therapy *n* = 90	*p*
Donor type, n (%)			0.821
Donation after brain death	75 (82.4%)	72 (80.0%)	
Donation after cardiac death	16 (17.6%)	18 (20.0%)	
Double kidney transplant, n (%)	19 (20.9%)	18 (20.0%)	1.000
Surgery length—min, IQR	225 [178–293]	253 [187–315]	0.132
Intraoperative vasopressors, n (%)	18 (19.8%)	16 (17.8%)	0.877
Total intraoperative fluids— mL, IQR	2000 [1500–2250]	1775 [1500–2475]	0.869
Intraoperative fluids/kg—mL/Kg/h, IQR	6.65 [4.23–9.63]	6.41 [4.67–9.05]	0.624
Intraoperative crystalloids—mL, IQR	2000 [1500–2250]	1750 [1500–2475]	0.882
Intraoperative colloids–—mL, IQR	0 [0–0]	0 [0–0]	0.308
Intraoperative blood mL, IQR	0 [0–0]	0 [0–0]	0.408
Total postoperative fluids— mL, IQR	2000 [1500–2500]	2000 [1500–2500]	0.959
Postoperative crystalloids—mL, IQR	2000 [1500–2500]	2000 [1500–2500]	0.894
Postoperative blood mL, IQR	0 [0–0]	0 [0–0]	0.190
Delayed graft function, n (%)	46 (50.5%)	50 (55.6%)	0.599
Pulmonary complications—5d, n (%)	25 (27.5%)	18 (20.0%)	0.314
Cardiovascular complications—5d, n (%)	8 (8.8%)	7 (7.8%)	1.000
Infectious complications—5d, n (%)	29 (31.9%)	33 (36.7%)	0.601
Any complication grade ≥ 3—30d, n (%)	47 (51.6%)	39 (43.3%)	0.331
Length of ICU stay—d, IQR	1 [1]	1 [0–1]	0.931
Length of hospital stay—d, IQR	**18 **[13–25]	**18 **[13–24]	**0.704**

*IQR* interquartile range, *ICU* intensive care unit

**Fig. 3:**
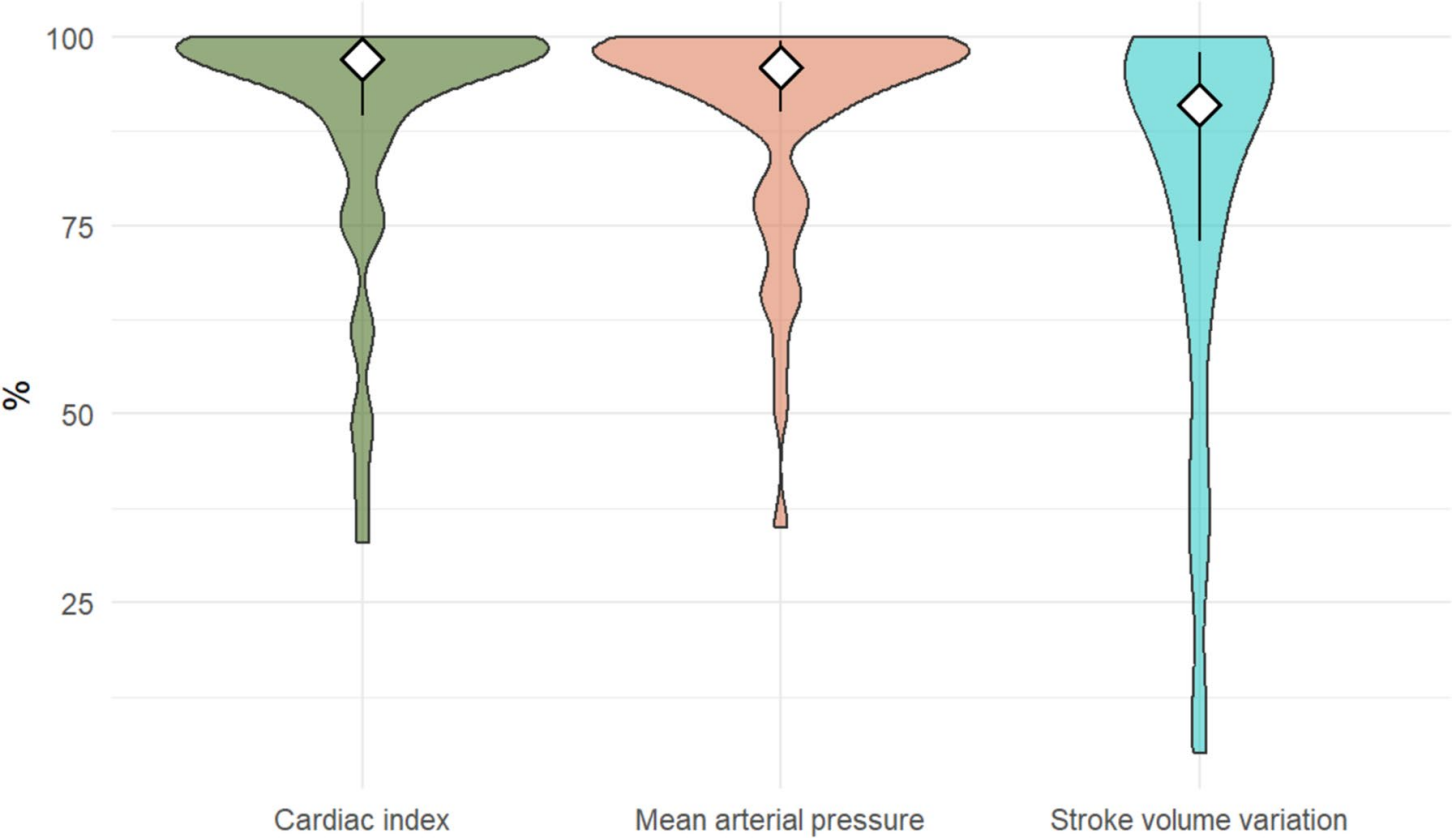
Compliance with goal-directed therapy protocol. Notes: this violin plot illustrates the distribution of the percentage of intraoperative time in which the target thresholds were satisfied. The parameters considered in the algorithm were mean arterial pressure (MAP), cardiac index (CI), and stroke volume variation (SVV). The violin shape represents the density of AIS values, with wider sections indicating higher frequency. The white diamond represents the median value and the whiskers the 25–75 interquartile range

Adherence data were available for 55 out of 91 patients (60.4%) in the GDT group. Overall, MAP exceeded the target threshold for 96% of the time [90.0%—99.5%], while CI and SVV met the target for 97% [89.5%—100%] and 91% [73%—98%] of the time, respectively (Fig. 3).

The length of postoperative ICU stay was identical, with a median of 1 day (IQR 1–1) in the GDT group and 1 day (IQR 0–1) in the conventional therapy group (*p* = 0.931).

The length of hospital stay, the primary outcome of this study, also did not differ significantly, with a median of 18 days (IQR 13–26) in the GDT group and 18 days (IQR 13–24) in the conventional fluid group (*p* = 0.704. The median difference was 0.5 days with a 95% confidence interval ranging from − 2.5 to 5 days.

**Fig. 4:**
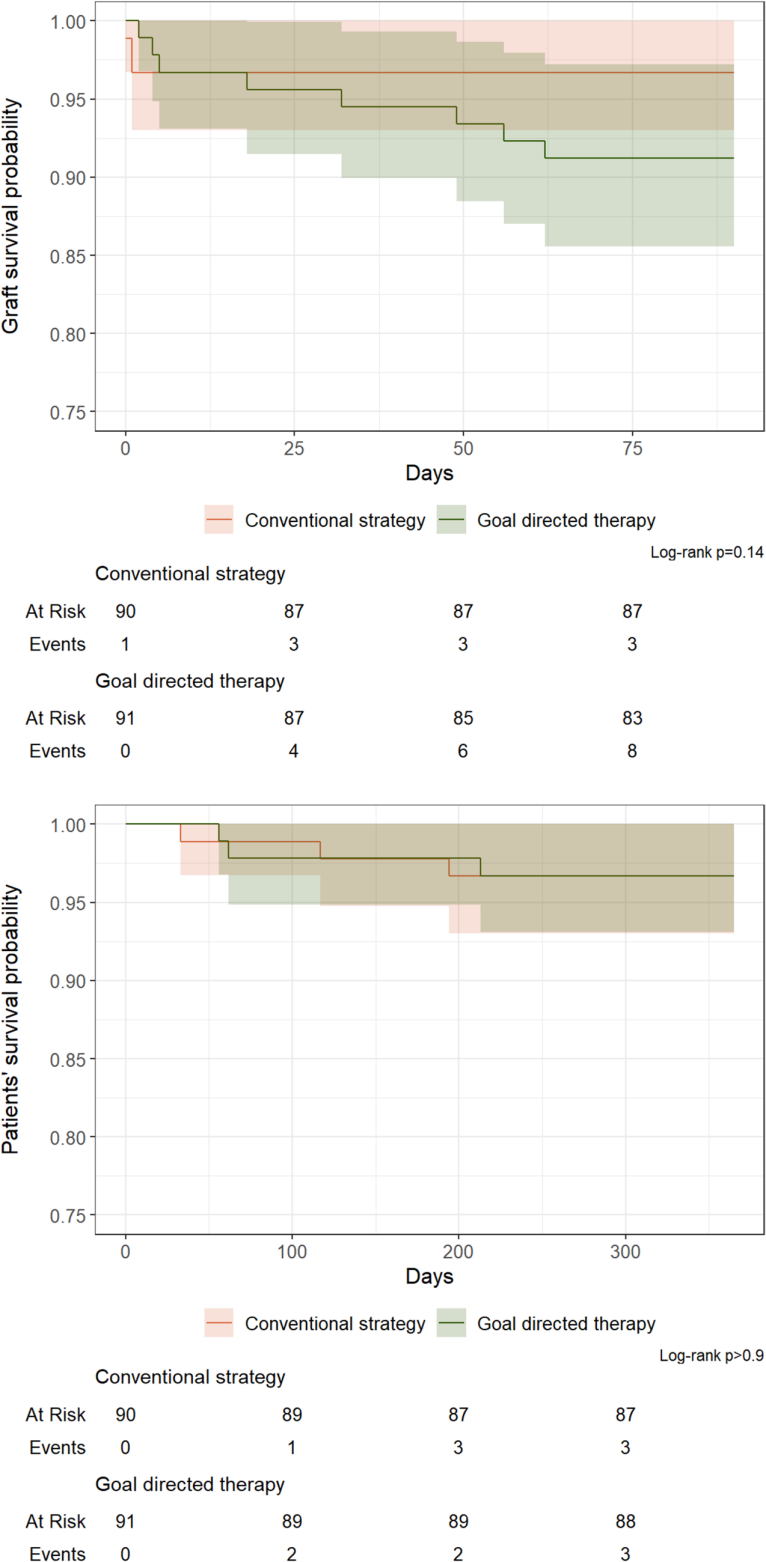
Kaplan–Meier survival curve for 90-day graft survival and 1-year patient survival

The 90-day graft survival and 1-year patient survival curves (Fig. 4) showed no significant differences between the two groups (log-rank test *p* = 0.140 and *p* > 0.9, respectively). Postoperative outcomes also revealed no significant differences between the groups. DGF occurred in 46 (50.5%) patients in the GDT group versus 50 (55.6%) in the conventional therapy group (*p* = 0.599). Complications within 30 days occurred in 47 (51.6%) patients in the GDT group and 39 (43.3%) in the conventional fluid group (*p* = 0.331).

In summary, there were no statistically significant differences in the primary and secondary outcomes between the goal-directed and conventional fluid management strategies in patients undergoing KT.

## Discussion

Goal-directed therapy has become increasingly popular in major surgeries, but its effectiveness in KT recipients remains unclear. This RCT aimed to determine whether GDT could reduce hospital length of stay, postoperative complications, DGF, 90-day graft survival, and 1-year patient survival in KT recipients. However, the study found no significant differences in any of these outcomes.

These findings are consistent with other RCTs on this topic [11, 13, 20, 21]. Several factors should be considered when interpreting these results.

First, GDT did not significantly change the volume of fluids administered during surgery and in the early postoperative period. This study took place in three different transplantation centers with dedicated anesthesiology teams. Adequate preoperative management and clinical expertise leading to an appropriate interpretation of available indices in the control group could partially explain the similar fluid management behavior between the two groups.

Comparing this study with other research, non-invasive GDT based on plethysmograph variability index and pulse pressure variation did not significantly affect the incidence of DGF, renal function tests, or urine output compared to conventional strategies [10, 20]. However, a lower intraoperative fluid administration was observed in the experimental groups [20].

TED monitoring is being evaluated for GDT guidance. A recent proof-of-concept trial found no differences in the volume of fluid administered or the incidence of complications. However, the study aimed to estimate a larger study sample size; hence, these results are not definitive [13]. TED was also recently compared with arterial waveform analysis, but no differences were found in fluid administration or postoperative complications [21].

A meta-analysis combining the results from available RCTs failed to demonstrate any differences in the incidence of DGF [22]. Consistently, animal models suggested that GDT did not improve early glomerular filtration rate, but may reduce tissue inflammation and preserve glycocalyx compared to high-volume fluid therapy [23].

On the other hand, some recent observational studies found a significant reduction in postoperative complications [8], and earlier mobilization [24] in patients exposed to GDT. We have to consider that in the latter study, fluid administration was reduced by about 30% in the group following GDT, suggesting a potential effect in real-life scenarios where conventional fluid administration results in excessive loads, as previously reported for animal models.

The lower effectiveness of GDT in KT compared to its general benefits in non-cardiac surgery [25] may be due to the different reliability of hemodynamic measurements in chronic kidney disease patients [18], and different non-invasive techniques could show different performance in these patients.

On the other hand, cannulating the radial artery in patients who are candidates for chronic renal replacement therapy poses risks that could compromise future radiocephalic fistula surgery, including temporary occlusion, sepsis, pseudoaneurysm, and permanent ischemic damage [26]. Hence, the use of non-invasive cardiac output monitoring techniques can help reduce the necessity for arterial cannulation while ensuring effective cardiovascular monitoring.

### Limitations

The results of this study may have been influenced by the fact that the sample size was not fully met (91% of the expected based on sample size calculation) and the study was conducted in high-volume transplantation centers with dedicated anesthesiology services.

Additionally, outcomes such as delayed graft function could be affected by various factors, including immunosuppression and vascular complications, which might have partially masked any differences.

Another consideration is that applying GDT protocols exclusively during the relatively short operative phase may have limited exposure time and impacted the outcomes.

However, it is essential to note that this is the first multicenter RCT exploring the effects of GDT in KT patients, and its sample size is significantly larger than that of previously published studies in this area [10, 11, 13, 20, 21].

## Conclusion

This RCT found no significant benefits associated with non-invasive pulse contour-based intraoperative GDT in terms of hospital length of stay, postoperative graft function, and surgical complications in patients undergoing KT.

Future research should investigate whether the non-invasive technology or the GDT protocol influenced the trial results. The DGF, which affected 50% of participants in the study, remains a significant postoperative concern in kidney transplants. Extending hemodynamic optimization beyond the intraoperative period could potentially enhance graft function, and non-invasive technology offers promising advantages in this context.

## Data Availability

Vitale, G. (2025): Impact of Intraoperative Goal-Directed Therapy on Perioperative Outcomes in Kidney Transplantation: A Multicenter Randomized Controlled Trial [Data set]. Zenodo. https://doi.org/10.5281/zenodo.14634807

## Abbreviations

ASA: American Society of Anesthesiologists
BIS: Bispectral index
BMI: Body mass index
CI: Cardiac index
CO: Cardiac output
COVID-19: Coronavirus disease 2019
CVP: Central venous pressure
DGF: Delayed graft function
DO_2_I: Oxygen delivery index
ETCO_2_: End-tidal carbon dioxide
FiO_2_: Fraction of inspired oxygen
GDT: Goal-directed therapy
Hb: Hemoglobin
Hct: Hematocrit
IBP: Invasive blood pressure
ICU: Intensive care unit
IQR: Interquartile range
KT: Kidney transplantation
MAC: Minimum alveolar concentration
MAP: Mean arterial pressure
NIBP: Non-invasive blood pressure
PEEP: Positive end-expiratory pressure
RBC: Red blood cells
RCT: Randomized controlled trial
SD: Standard deviation
SpO2: Peripheral oxygen saturation
SV: Stroke volume
SVR: Systemic vascular resistance
SVV: Stroke volume variation
TAP: Transversus abdominis plane
TED: Transesophageal Doppler
